# Identifying Highly Penetrant Disease Causal Mutations Using Next Generation Sequencing: Guide to Whole Process

**DOI:** 10.1155/2015/923491

**Published:** 2015-04-06

**Authors:** A. Mesut Erzurumluoglu, Santiago Rodriguez, Hashem A. Shihab, Denis Baird, Tom G. Richardson, Ian N. M. Day, Tom R. Gaunt

**Affiliations:** ^1^Bristol Genetic Epidemiology Laboratories (BGEL), School of Social and Community Medicine, University of Bristol, Oakfield House, Oakfield Grove, Bristol BS8 2BN, UK; ^2^MRC Integrative Epidemiology Unit (IEU), School of Social and Community Medicine, University of Bristol, Oakfield House, Oakfield Grove, Bristol BS8 2BN, UK

## Abstract

Recent technological advances have created challenges for geneticists and a need to adapt to a wide range of new bioinformatics tools and an expanding wealth of publicly available data (e.g., mutation databases, and software). This wide range of methods and a diversity of file formats used in sequence analysis is a significant issue, with a considerable amount of time spent before anyone can even attempt to analyse the genetic basis of human disorders. Another point to consider that is although many possess “just enough” knowledge to analyse their data, they do not make full use of the tools and databases that are available and also do not fully understand how their data was created. The primary aim of this review is to document some of the key approaches and provide an analysis schema to make the analysis process more efficient and reliable in the context of discovering highly penetrant causal mutations/genes. This review will also compare the methods used to identify highly penetrant variants when data is obtained from consanguineous individuals as opposed to nonconsanguineous; and when Mendelian disorders are analysed as opposed to common-complex disorders.

## 1. Introduction

Next generation sequencing (NGS) and other high throughput technologies have brought new challenges concomitantly. The colossal amount of information that is produced has led researchers to look for ways of reducing the time and effort it takes to analyse the resulting data whilst also keeping up with the storage needs of the resulting files, which are in the magnitude of gigabytes each. The recently emerged variant call format (VCF) has somewhat provided a way out of this complex issue [1]. Using a reference sequence and comparing it with the query sequence, only the differences (i.e., variants) between the two are encoded into a VCF file. Not only are VCF files substantially smaller in size (e.g., for whole-exome data, <300x in relation to BAM files which store all raw read alignments), they also make the data relatively easy to analyse since there are many bioinformatics tools (e.g., annotation and mutation effect prediction) which accept the VCF format as standard input. The genome analysis toolkit (GATK) made available by the Broad Institute also provides useful suggestions to bring a universal standard for the annotation and filtering of variants in VCF files [2]. The abovementioned reasons have made the VCF the established format for the sharing of genetic variation produced from large sequencing projects (e.g., 1000 Genomes Project, NHLBI Exome Project, also known as EVS). However the VCF does have some disadvantages. The files can be information dense and initially difficult to understand and parse. Comprehensive information about the VCF and its companion software VFCtools [1] is available online (http://vcftools.sourceforge.net/).

Because of the substantial decrease in the price of DNA sequencing and SNP chip arrays [3], there has been a sharp increase in the number of genetic association studies being carried out, especially in the form of genome-wide association studies (GWAS, statistics available at http://www.genome.gov/gwastudies/). As whole genome sequencing (WGS) is prohibitively expensive for large genetic association studies [4–6], whole exome sequencing (WES) has emerged as the attractive alternative, where only the protein coding region of the genome (i.e., exome) is targeted and sequenced [7]. The decision to carry out WES over WGS is not solely influenced by the cost which currently stands at one-third in comparison [8], but also by the fact that most of the known Mendelian disorders (~85%) are caused by mutations in the exome [9]; and reliably interpreting variation outside of the exome is still challenging as there is little consensus on interpreting their functional effects (even with ENCODE data [10] and noncoding variant effect prediction tools such as CADD [11], FATHMM-MKL [12], and GWAVA [13]). For complex diseases, WES can provide more evidence for causality compared to GWAS, assuming that the causal variants are exonic. This is because the latter uses linkage disequilibrium (LD) patterns between common markers [14] whereas WES directly associates the variant itself with the trait/disorder. Therefore using GWAS, especially in gene-dense regions, one cannot usually make conclusive judgements about which gene(s) is causal without further sequencing or functional analysis. WES has been successfully used in identifying and/or verifying over 300 causal variants for Mendelian disorders (statistics from http://omim.org/) (also see references [15, 16] for discussion of the use and benefits of WES in clinical genetics). WES currently stands at approximately $1000 for 50x read depth (variable prices, less for larger studies). However since there is a great deal of variation in the human genome [17], finding the causal variant(s), especially ones with low penetrance, is not going to be trivial. This problem can be exacerbated by the nature of the disorder(s) analysed. It is relatively easier to map variants causing rare monogenic diseases (when several affected individuals/families are available for analysis), as there is most likely to be a single variant present in the cases that is not in the controls; but in contrast, common complex (polygenic) disorders are much harder to dissect when searching for causal variants.

In this paper, our aims are to (i) provide a guide for genetic association studies dealing with sequencing data to identify highly penetrant variants (ii) compare the different approaches taken when data is obtained from unrelated or consanguineous individuals, and (iii) make suggestions about how to rank single nucleotide variation (SNV) and/or insertion/deletions (indels) following the standard filtering/ranking steps if there are several candidate variants, using annotated variants within VCF files as examples. To aid the process of analysing sequencing data obtained from consanguineous individuals, we have also made available an autozygosity mapping algorithm (AutoZplotter) which takes VCF files as input and enables manual identification of regions that have longer stretches of homozygosity than would be expected by chance.

## 2. Stage 1: Quality Control and Variant Calling

Before any genetic analysis, it is important to understand how the raw data were produced and processed to make better judgements about the reliability of the data received. Thorough quality control steps are required to ensure the reliability of the dataset. Lack of adequate prior quality control will inevitably lead to loss of statistical power and increase false positive and false negative findings. Fully comprehending each step during the creation of the dataset will have implications on the interpretation stage, where genotyping errors (also known as “phantom” mutations [18]) may turn out to be statistically associated (e.g., batch effects between case and control batches) or the causal variant may not be identified due to poorly applied quality control (QC) and/or filtering methods. The most fitting example for this comes from a recent primary ciliary dyskinesia (PCD) study [19], where the causal variant was only detected after the authors manually noticed an absence of reads in the relevant region of the genome (personal communication with authors). The subsequent variant was not only missing in the VCF files, but also in the initial BAM files, requiring remapping of reads. Another point of consideration from this finding would be that the authors knew where to look because the* RSPH9* gene (the p.Lys268del mutation) was one of their* a priori* candidates [20]. This is also an example demonstrating the importance of deep prior knowledge and screening for known variants as it is impossible for one to manually check the whole exome (or the genome) for sequencing and/or mapping errors.

### 2.1. Targeted Sequencing

**Figure 1:**
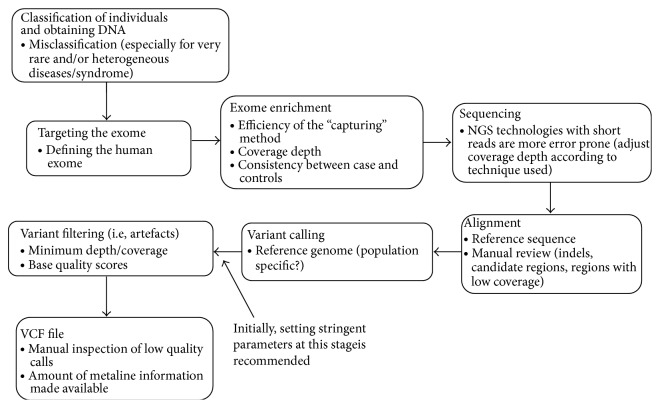
Steps in whole-exome sequencing. Understanding how the VCF file was created is important, as it can give an idea about where something may have gone wrong. The stages proceed from top to bottom and we have proposed “consideration points” for each step (below each title).

As far as WES projects are concerned, questions about coverage arise right from the start (Figure 1). Since knowledge concerning exons in our own genome is far from complete, there are differing definitions about the human exome coordinates. Therefore, the targeted regions by the commonly used and commercially available Agilent SureSelect [21] and the Nimblegen SeqCap EZ [22] exome capture kits are not entirely overlapping [23]. Thus it is possible that the missing regions of the exome due to the chosen probe kit may turn out to have the functional region in relation to the disorder analysed. One must also bear in mind that the kits available for targeting the exome are not fully efficient due to a certain quantity of poorly synthesized and/or designed probes not being able to hybridize to the target DNA. Next step is target enrichment where high coverage is vital as NGS machines produce more erroneous base calls compared to other techniques [24]; therefore, especially for rare variant analyses, it is important to have data with high average read depth (i.e., ≥50x).

### 2.2. Mapping Sequence Reads

The raw reads produced should then be aligned to a reference genome (e.g., GRCh38, see NCBI Genome Reference Consortium) and there are many open sources and widely applied tools (Table 1). However, solely depending on automated methods and software can leave many reads spanning insertions and deletions (indels) misaligned; therefore postreviewing the data for mismapping is always a good practice, especially in the candidate regions. Attempting to remap misaligned reads with a lower stringency using software such as Pindel would be an ideal way to go about solving such a problem [25]. GATK also provides a base recalibration and indel realignment algorithm for this purpose.

Effective variant calling depends on accurate mapping to a dependable reference sequence. If available, using a population specific reference genome would be most ideal to filter out known neutral SNPs existing within the region of origin of the analysed subjects (e.g., East-Asian reference genome for subjects of Japanese origin). Inclusion of ambiguity codes (e.g., IUPAC codes) for known polyallelic variants to create a composite reference genome can also be useful (although not essential).

### 2.3. Variant Calling

There are many tools available for the identification of SNVs, indels, splice-site variants, and CNVs present in the query sequence(s). Each variant calling tool has advantages and disadvantages and has made compromises relating to issues such as speed of analysis, annotation, and reliability of the output file (Table 2). Separating true variation from sequencing artefacts still represents a considerable challenge. When dealing with very rare disorders, the candidate regions in the output VCF (or BAM) files should be reviewed either by reviewing the QC scores in the VCF or by visualising the alignments in IGV [26]. Performing this step could highlight sequencing errors such as overcoverage (due to greater abundance of capture probes for the region or double capturing due to poorly discriminated probes hybridising to the same region) or undercoverage (due to probes not hybridising because of high variability in the region). For rare Mendelian disorders, since there is going to be a single causal variant it is more important to make sure that the variants in the dataset are reliable. Therefore setting strict parameters for read depth (e.g., ≥10x), base quality score (e.g., ≥100), and genotype quality scores (e.g., ≥100) initially can eliminate wrong base and genotype calls. This can then be adjusted subsequently (i.e., made less stringent) if no variants with a strong candidacy for causality are found after filtering (also see Best Practices section of GATK documentation for variant analysis).

As mentioned above, there are many tools available for the identification of variants present in the query sequence (see Table 2). GATK [2] is one of the most established SNP discovery and genome analysis toolkits, with extensive documentation and helpful forums. It is a structured programming framework which makes use of the programming philosophy of MapReduce to solve the data management challenge of NGS by separating data access patterns from analysis algorithms. GATK is constantly updated and cited and also has a vibrant forum which is maintained continually.

SAMtools [27] is a variant caller which uses a Bayesian approach and has been used in many WGS and WES projects including the 1000 Genomes Project [17]. SAMtools also offers many additional features such as alignment viewing and conversion to a BAM file. A recent study has compared GATK, SAMtools, and Atlas2 and found GATK to perform best in many settings (see reference [28] for details). However all three were highly consistent with an overlapping rate of ~90%. SOAPsnp is another highly used SNP and genotype caller and is part of the reliable SOAP family of bioinformatics tools (http://soap.genomics.org.cn/).

### 2.4. Additional Checks of Autozygosity

For data obtained from consanguineous families, confirming expected autozygosity (i.e., homozygous for alleles inherited from a common ancestor) would be an additional check worth carrying out. If the individual is the offspring of first cousins then the level of autozygosity would be approximately 6.25% (*F* = 0.0625) and 12.5% (*F* = 0.125) for offspring of double first cousins (or uncle-niece unions, see Figure S1 in Supplementary Material available online at http://dx.doi.org/10.1155/2015/923491 for a depiction of these). These values will be higher in endogamous populations (e.g., for offspring of first cousins: 6.25% + autozygosity brought about due to endogamy, see Figure S3 for an example). Autozygosity could be checked by inspecting long runs of homozygosity (LRoH) for each individual by using tools such as Plink (for SNP chip data) [29], EXCLUDEAR (for SNP chip data) [30], AgilentVariantMapper (for WES data) [31], and AutoSNPa (for SNP chip data) [32] and dividing total autozygous regions by total length of autosomes in the human genome (can be obtained from http://www.ensembl.org/Homo_sapiens/Location/Genome). AutoZplotter (available to download in Supplementary Materials) that we developed takes VCF files as input, enabling easy and reliable visualisation and analysis of LRoH for any type of data (WGS, WES or SNP chip). The code (written in the Python programming language) can also be adapted relatively easily for use in analyses of other species.

## 3. Stage 2: Filtering/Ranking of Variants

Once the quality control process is complete and VCF files are deemed “analysis ready,” the approach taken will depend on the type of disorder analysed. For rare Mendelian disorders, many filtering and/or ranking steps can be taken to reduce the thousands of variants to a few strong candidates. Screening previously identified genes for causal variants is a good starting point. Carrying out this simple check will allow the identification of the causal variant even from a single proband thus saving time, effort, and funding. If no previously identified variant is found in the proband analysed, there are several steps which can be taken to identify novel mutations.

### 3.1. Using Prior Information to Rank/Filter Variants

Locus specific databases (see http://www.hgvs.org/dblist/glsdb.html for a comprehensive list) and “whole-genome” mutation databases such as HGMD [33], ClinVar [34], LOVD [35], and OMIM [36] are very informative resources for this task. Finding no previously identified variants indicates a novel variant in the proband analysed. For rare Mendelian disorders, the look for the variant can begin by removal of known neutral and/or common variants (≥0.1%) as this would provide a smaller subset of potentially causal variants. This is a pragmatic choice as Mendelian disease causal variants are likely to be very rare in the population or unique to the proband. If the latter is true, the variant should be absent from public databases. For this process to be thorough, an automated annotation tool such as Ensembl VEP or ANNOVAR can be used (see reference [37] for a review on the caveats of using these consequence predictors). Ensembl VEP enables incorporation of allele frequency (labelled as GMAF, global minor allele frequency) information from the EVS and the 1000 Genomes Project (see Supplementary Material and Methods for details).

### 3.2. Using Effect Prediction Algorithms to Rank/Filter Variants

**Figure 2:**
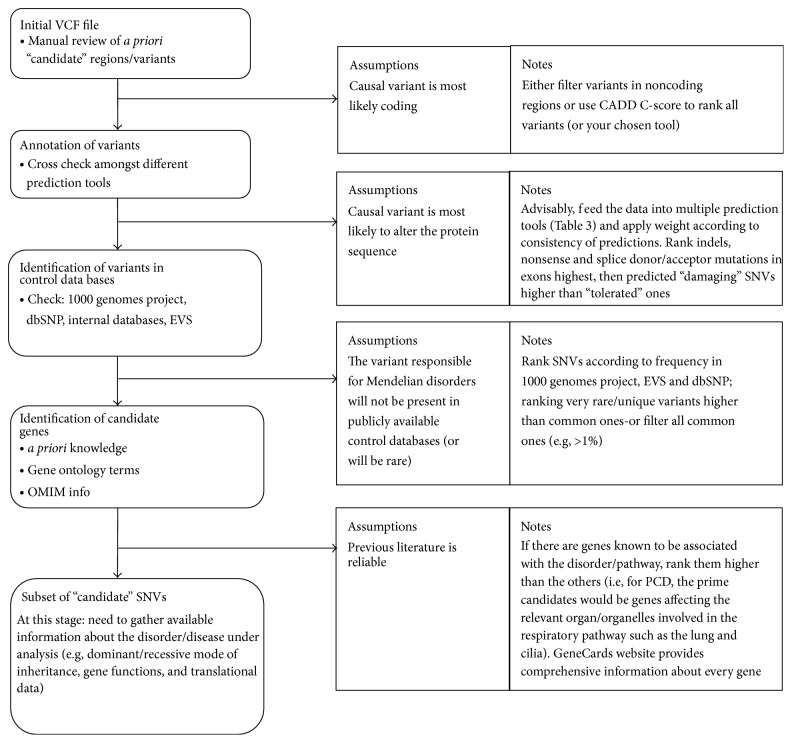
Post-VCF file procedures (example for sequencing data). Every step here can be automated through the use of pipelines and bioinformatics tools. Whilst performing the steps listed above, one must always bear in mind the assumptions behind the procedures. Where feasible, ranking of rare SNVs would be advised over filtering as it allows the researcher to observe all variants as a continuum from most likely to least likely.

Ranking this subset of variants based on consequence (e.g., stop gains would rank higher than missense) and scores derived from mutation prediction tools (e.g., “probably damaging” variants would rank higher than “possibly damaging” according to Polyphen-2 prediction) would enable assessment of the predicted impact of all rare mutations. It is important to understand what is assumed at each filtering/ranking stage; and comments are included about each assumption and their caveats in Figure 2.

For individuals of European ancestry, a VCF file will have between eighty and ninety thousand variants for WES (more for individuals with African ancestry [38]); and approximately a tenth will be variants with “predicted high impact” (also known as Φ variants, i.e., rare nonsense, missense, splice-site acceptor or donor variants, exonic indels, and start losses [39]). There are many algorithms which predict the functional effect of these variants (Table 3). A large proportion of these algorithms utilize sequence conservation within a multiple sequence alignment (MSA) of homologous sequences to identify intolerant substitutions, for example, a substitution falling within a conserved region of the alignment is less likely to be tolerated than a substitution falling within a diverse region of the alignment (see reference [40] for a review). A handful of these algorithms also utilize structural properties, such as the protein secondary structure and solvent accessible surface area, in order to boost performance. Well known examples of a sequence-based and structure-based algorithm are SIFT [41] and PolyPhen [42], respectively. Newer software such as FATHMM [43] and MutPred [44], which use state-of-the-art hidden Markov models and machine learning paradigms, are worth using for their performance. There are also several tools such as CONDEL-2 [45] which combine the output of several prediction tools to produce a consensus deleteriousness score. Although SIFT and Polyphen are highly cited tools, comparative analyses carried out by Thusberg et al. and Shihab et al. found FATHMM, MutPred, and SNPs&GO to perform better using the VariBench benchmarking dataset containing missense mutations [43, 46]. For predicting the effects of noncoding variants, FATHMM-MKL [12], GWAVA [13], and/or CADD [11] should be used. Also Human Splice Finder (latest: v3.0) can be used for intronic variants which predicts whether splicing is affected by the variant or not [47]. Many of these tools can be incorporated into the analyses through the Ensembl website (http://www.ensembl.org/info/docs/tools/vep/index.html) where VCF files are annotated [48].

These prediction algorithms are, as their name suggests, only there to make predictions about whether a variant is expected to be functionally disruptive or not. Thus their main purpose is to enable researchers to rank certain variants higher than others in order for them to be studied in a systematic way. Thus they do not “prove” anything about the causality of the variant. The variants predicted “deleterious” still require following up through replication and/or functional studies. Also disagreements amongst different tools can be observed which can lead to different interpretations about the evolutionary history of the variant (e.g., same function conserved throughout different species or a recently acquired function). Users of prediction algorithms should be aware of how these algorithms derive their predictions and then decide whether the tool can be generalized to their datasets. For example, those interested in somatic mutations should choose cancer-specific algorithms for example, FATHMM-Cancer [49] and SPF-Cancer [50], given that germline variant prediction algorithms are incapable of discriminating between cancer driver mutations and other germline mutations.

### 3.3. Further Filtering/Ranking

With current knowledge, there are approximately fifty synonymous mutations with proven causality, complex traits and Mendelian disorders combined [51]. This is a very small proportion when compared to the thousands of published clinically relevant nonsynonymous (i.e., missense and nonsense) mutations. Therefore, when filtering variants for rare monogenic disorders, not taking noncoding variants and synonymous variants into account in the initial stages is a pragmatic choice. If ranking is preferred, then tools such as SilVA [52], which ranks all synonymous variants, and CADD [11] which ranks all variants (including synonymous variants) in the VCF files should be used.

Highly penetrant (Mendelian or common-complex) disease causal variants are expected to be very rare; therefore most of them should not appear in publicly available datasets. However filtering all variants present in dbSNP which is common practice should not be carried out as amplification and/or sequencing errors as well as potentially causal variants are known to make their way into this database (see references [53, 54] for details). Thus use of a MAF threshold (e.g., ≤0.1% in 1000 genomes and/or EVS) is a wiser choice in contrast to using absence in dbSNP as a filter. Upon completion of these steps, a smaller subset of variants with strong candidacy will remain for further follow up to determine causality.

Another initially pragmatic choice is to filter out all the annotations except for the “canonical” transcripts (i.e., longest transcript of a gene, if several exist) as this can reduce the amount of variants present in the Ensembl VEP (or ANNOVAR) annotated files considerably (~5x fold). However, this can be a problem for genes where the canonical transcript does not contain all the exons present within the gene, as a mutation which falls in an exon which is not present in the canonical transcript will not be observed in the filtered file (coded “CANONICAL” in Ensembl VEP annotated variants).

As many online tools are expected to keep logs of the processes undergoing in their servers, to protect the confidentiality of genetic information, downloading a local version of the chosen tools (or the VEP cache from the Ensembl website) is recommended. VEP also enables the incorporation of many other annotations (e.g., conservation scores, is variant position present in HGMD public version, whether variant is cited in PubMed), which will make the screening and filtering steps more manageable.

## 4. Stage 3: Building Evidence for Causality

**Figure 3:**
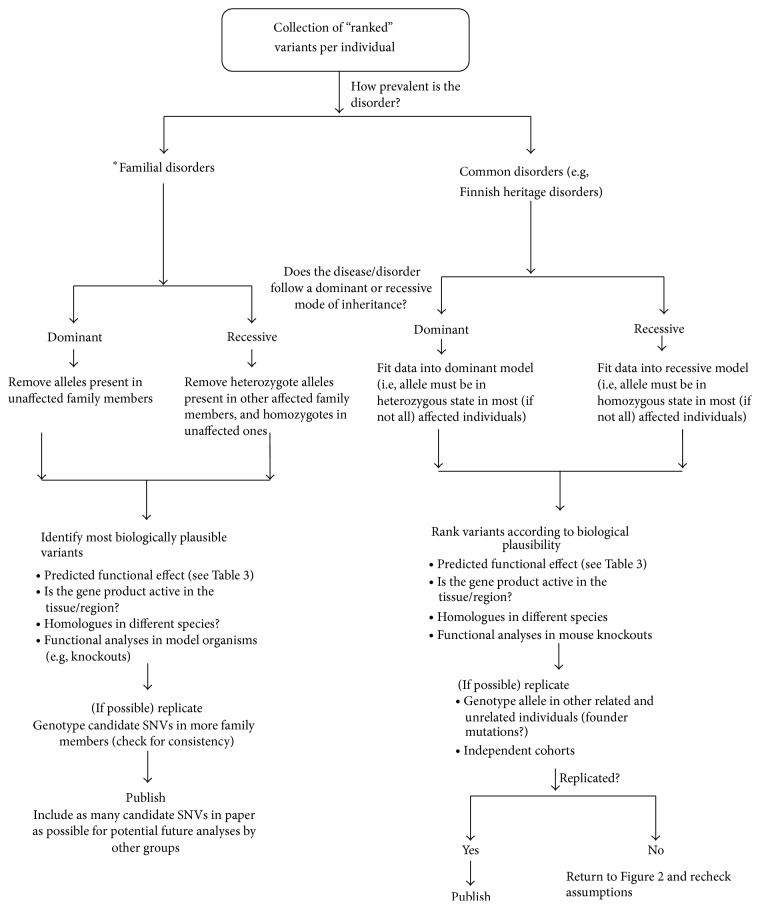
Finding “the one” in Mendelian disorders. Searching for the causal variant (using a WES example). After potentially causal variants are identified, one must put into practice what past literature suggests about the disorder and make certain decisions about which path to follow in Figure 3. Familial (very rare) disorders are more likely to be following a recessive mode of inheritance; thus family data is crucial (to rule out the possibility of* de novo* mutations). Also it is crucial to include as many family members as possible. For common Mendelian disorders, if the disorder is following a recessive inheritance model, the possibility of the existence of compound heterozygotes should be taken into account when fitting the data into a recessive model. Finally, functional postanalysis of candidate variant(s), especially in mouse knockouts, can be crucial. This figure is here to serve as an example and by no means reflects an exhaustive model; there are alternative routes that researchers can take to identify Mendelian causal variants. ^∗^If a consanguineous family, identifies regions where there are long runs of homozygosity (LRoH) for each individual, and amongst these regions, the ones which are shared by the affected and not by the unaffected.

Figure 3 suggests an example route to take to help differentiate causal variant(s) from noncausal ones for Mendelian disorders. At this stage one must gather all information that is available about the disorder and use it to determine which inheritance pattern fits the data and what complications there might be (e.g., the possibility of compound heterozygotes in disorders which show allelic heterogeneity). Figure S2 can be used to observe the contrast between the routes taken when analysing Mendelian (Figure 3) and complex disorders.

### 4.1. Public Data as a Source of Evidence

Having a candidate gene list based on previously published literature (e.g., by using OMIM or a disease/pathway specific database such as the Ciliome database [55]) and knowledge about the biology of the disorder (e.g., biological pathways) is useful. Software such as STRING and KEGG predicts protein-protein interactions using a variety of sources [56, 57]. SNPs3D has a user friendly interface which is designed to suggest candidates for different disorders [58]. UCSC Gene Sorter (accessible from https://genome.ucsc.edu/) is another useful tool for collating a candidate gene list as it groups genes according to several features such as protein homology, co-expression and gene ontology (GO) similarity. Uniprot's (http://www.uniprot.org/) Blast and Align functions can provide essential information about the crucial role a certain residue plays within a protein if it is highly conserved throughout many species. This is especially important for SNVs where the SNV loci itself should be causal (e.g., missense mutations, excludeing nonsense mutations as they truncate the gene product, thus the deleted segment of the protein requires further follow-up to prove causality, not just the loci where the mutation occurred as in other SNVs).

An example of the filtering process for an autosomal recessive disorder such as PCD is depicted in Figure 5. If several variants pass the filtering steps, information about the relevant genes should be gathered using databases such as GeneCards (http://www.genecards.org/) and NCBI Gene (http://www.ncbi.nlm.nih.gov/gene) for functional information, GEO Profiles (http://www.ncbi.nlm.nih.gov/geoprofiles) and Unigene (http://www.ncbi.nlm.nih.gov/unigene) for translational data about the gene's product; and if available, one can check if a homologue is present in different species using databases such as HomoloGene (http://www.ncbi.nlm.nih.gov/homologene) and whether a similar phenotype is observed in model organisms. For example, if the disorder affects the cerebral cortex but the gene product is only active in the tissues located in the foot, then one cannot make a good argument about the identified variant in the respective gene as being “causal.”

There are many complications that may arise depending on the disorder such as genetic (locus) heterogeneity [59], allelic heterogeneity [60] and incomplete penetrance [61]. Therefore gathering as many cases from the same family is helpful. However for very rare Mendelian disorders this may not be possible, thus it is important to seek other lines of evidence for causality (e.g., animal models, molecular analyses).

### 4.2. Mapping Causal Loci within Families

For rare Mendelian disorders, familial information can be crucial. The availability of an extended pedigree can be very informative in mapping which variant(s) fits the mode of inheritance in the case(s) and not in the unaffected members of the family (e.g., for autosomal recessive mutations, confirming heterozygosity in the parents is a must). This will provide linkage data where its importance is best displayed by Sobreira et al. where WES data from a single proband was sufficient in discovering the causal variants in two different families [62]. Where available, previously published linkage data (i.e., associating a chromosomal region to a Mendelian disorder) should also made use of.

Traditionally a LOD score of 3 (Prob. = 1/1000) is required for a variant/region to be accepted as causal. Reaching this threshold requires many large families with many affected individuals. However this is not feasible for most disease causal variants (which are very rare by nature) and other lines of evidence such as animal knockouts, molecular studies and local sequence alignments (by using UniProt as mentioned above) are required to make a case for the causality of variants, especially mutations which are not stop gains (e.g., missense).

As mentioned previously, understanding the characteristics of a Mendelian disorder is important. If the disorder is categorised as “familial” (i.e., occurs more in families than by chance alone), which are usually very rare by nature, then availability of familial data becomes crucial, as unaffected members of the family are going to be the main source of information when determining neutral alleles. Any homozygous (and rare) stop gains, splice-site acceptor/donor variants and start losses in previously identified genes would be prime candidates.

Approach taken in families is different from the approaches taken when analysing common Mendelian disorders using unrelated individuals. For common Mendelian disorders (e.g., Finnish Heritage disorders [63–65]), fitting the dataset into a recessive inheritance model requires most (if not all) affected individuals to have two copies of the disease allele, enabling the identification of founder mutations as they will be overrepresented in the cases. These variants will be homozygous through endogamy and not consanguinity.

### 4.3. Autozygosity Mapping

For consanguineous subjects, the causal mutation usually lies within an autozygous region (characterised by long regions of homozygosity, LRoH, which are generally >5 Mb, see [66]), thus checking whether any candidate genes overlaps with an LRoH can narrow region(s) of interest. There are several tools which can identify LRoHs such as Plink, AutoSNPa and AgilentVariantMapper. We have made available a user-friendly python script (AutoZplotter) to plot heterozygosity/homozygosity status of variants in VCF files to allow for manual screening of short autozygous regions as well as LRoHs.

### 4.4. AutoZplotter

There are several software which can detect long runs of homozygosity reliably (>5 Mb), however they struggle to identify regions that are shorter. Therefore we developed AutoZplotter which plots homozygosity/heterozygosity state and enables quick visualisation of suspected autozygous regions (requires Xming or other X11 display server). These regions can then be followed up in more detail if any overlaps with a candidate gene/region. The input format of AutoZplotter is VCF thus it suits any type of genetic data (e.g., SNP array, WES, WGS). AutoZplotter was used for this purpose in a previous study by Alsaadi et al. [19].

### 4.5. Exceptional Cases

There can always be exceptional cases (in consanguineous families also) such as compound heterozygotes (i.e., individuals carrying different variants in the two copies of the same gene). This would require haplotype phasing and the confirmation of variant status (i.e., heterozygosity for one allele and absence of the other) in the parents and the proband(s) by sequencing of PCR amplicons containing variant or genotyping the variant directly. Beagle and HAPI-UR are two widely used haplotype phasing tools for their efficiency and speed [67, 68].

### 4.6. Identifying Highly Penetrant Variants for Common-Complex Disorders

For common complex disorders, identifying causal variants in outbred populations has proven to be a difficult and costly process (Supp. Figure S2); and these disorders can have many unknowns such as the significance of environmental factors on the disorder (see two examples of differential environmental influence on disease/traits in references [69, 70]) and epistasis [71]. Many of the causal variants may be relatively rare (and almost always in heterozygous state) in the population introducing issues with statistical power. Traditional GWAS do not attempt to analyse them thus they are largely ignored, leaving a lot of heritability of common complex disorders unexplained. Analysing individuals with extreme phenotypes where the segregation of disease mimics autosomal recessive disorders (e.g., in consanguineous families) can be useful in identifying highly penetrant causal genes/mutations for complex disorders (e.g., obesity and leptin gene mutations [72]). The genetic influence in these individuals is predicted to be higher and is expected to have a single highly penetrant variant in homozygous state. These highly penetrant mutations can mimic Mendelian disorders causal variants. Therefore similar study designs can be used as stated above (e.g., Autozygosity/homozygosity mapping).

## 5. Conclusions

**Figure 4:**
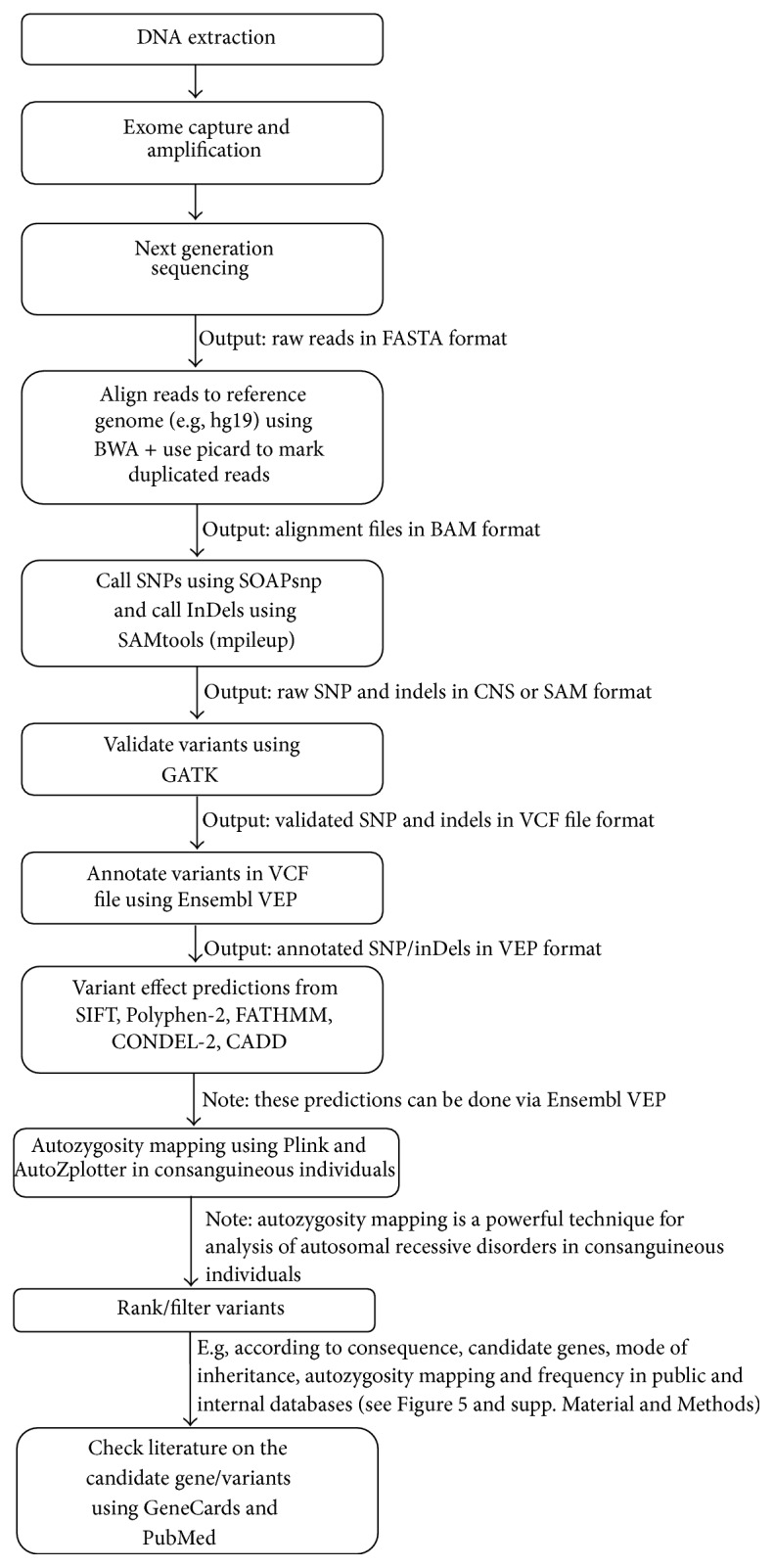
Summary of whole analysis process. DNA sample to identification of variant. The tools mentioned here are the ones we prefer to use for a variety of reasons such as having user-friendly documentation, ease of use, performance, multiplatform compatibility, and speed. See Supplementary Material and Methods for examples of parameters/commands to use where applicable.

**Figure 5:**
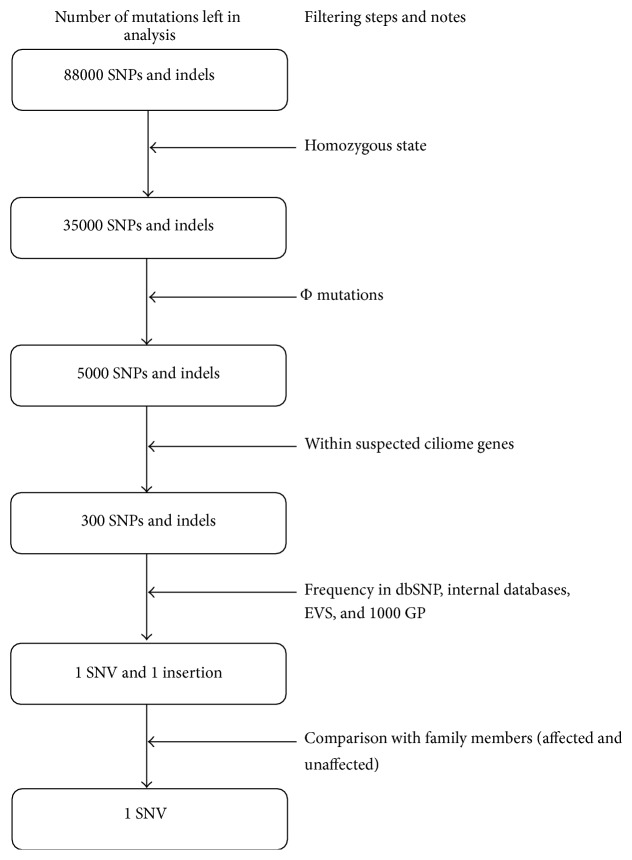
Filtering steps applied to all mutations in the exome (primary ciliary dyskinesia example). After all the filtering steps in the above figure are applied, the total will be reduced to a single candidate. The numbers here are for illustration purposes only (adapted from [39]). Homozygosity step is added as PCD is an autosomal recessive disorder. Φ mutations are “predicted high impact” mutations as proposed by Alsaadi et al. [39] (see PHI_SO_terms.txt in Supplementary data).

The NGS era has brought data management problems to traditional geneticists. Many data formats and bioinformatics tools have been developed to tackle this problem. One can easily be lost in the plethora of databases, data formats and tools. “Which tools are out there? How do I use it? What do I do next with the data I have?” are continually asked questions. This review aims to guide the reader in the rapidly changing and ever expanding world of bioinformatics.  Figure 4 depicts a summary of the analysis process from DNA extraction to finding the causal variant, putting into perspective which file formats are expected at each step and which bioinformatics tools we prefer due to reasons mentioned before. Researchers can then appreciate the stage that they are at and how many other steps are required for completion as well as knowing what to do at each step.

Whole exome sequencing is the current gold standard in the discovery of highly penetrant disease causal mutations. As knowledge on the noncoding parts of the genome can still be considered to be in its early days, the human exome is still a pragmatic target for many. As approx. 1600 known Mendelian disorders (and ~3500 when suspected ones are included) and most common-complex disorders are still waiting for their molecular basis to be figured out (from http://omim.org/statistics/entry, true as of 15/07/14), future genetic studies have much to discover. However for these projects to be fruitful, careful planning is needed to make full use of available tools and databases (see Table 4).

Finally, with this paper we have also made AutoZplotter available (input format: VCF), which plots homozygosity/heterozygosity state and enables quick visualisation of suspected autozygous regions. This can be important for shorter autozygous regions where other autozygosity mappers struggle.

## Supplementary Material

S1: List of useful bioinformatics command, parameter and files for variant calling, annotation and analysis.
Figure S1: Pedigree trees demonstrating examples of relevant consanguineous unions.
Figure S2: Example of the routes taken when analysing complex disorders.

## Figures and Tables

**Table 1:** Tools for aligning reads to a reference genome.

Name	References	Comment
BFAST Bowtie 2	[73] [74]	These aligners use similar algorithms to determine contiguous sequences; however MAQ and BWA are widely used and have been praised for their computational efficiency and multiplatform compatibility [75].
BWA MAQ SOAP2	[76] [77] [78]	

These are some of the many tools built for aligning reads produced from high throughput sequencing. Some have made speed their main purpose whereas others have paid more attention to annotating the files produced (such as mapping quality).

**Table 2:** Tools for identifying variation from a reference genome using NGS reads.

Name	References	URL	Comment
GATK	[2]	http://www.broadinstitute.org/gatk/	(i) Arguably the most established genome analysis toolkit (ii) Includes tools such as Unified Genotyper (SNP/genotype caller), variant filtration (for filtering SNPs), and variant Recalibrator (for SNP quality scores) (iii) Well documented with forums (iv) Input: SAM format (v) Output: VCF format

QCALL	[79]	ftp://ftp.sanger.ac.uk/pub/rd/QCALL	(i) Theoretically calls “high quality” SNPs even from low-coverage sequencing data (ii) Makes use of linkage disequilibrium information

PyroBayes	[80]	http://bioinformatics.bc.edu/marthlab/wiki/index.php/PyroBayes	(i) Theoretically makes “confident” base calls even in shallow read coverage for reads produced by Pyrosequencing machines.

SAMTools	[27]	http://samtools.sourceforge.net/	(i) Computes genotype likelihoods (ii) BCFtools calls SNP and genotypes (iii) Successfully used in many WGS and WES projects such as the 1000 Genomes Project [17]. (iv) Offers additional features such as viewing alignments and conversion of SAM to a BAM format

SOAPsnp	[81]	http://soap.genomics.org.cn/soapsnp.html	(i) Part of the reliable SOAP family of bioinformatics tools (ii) Well-documented website and cited and used by many [82, 83].

Control-FREEC	[84]	http://bioinfo-out.curie.fr/projects/freec/	(i) Identifies copy number variations (CNVs) between case and controls from sequencing data (ii) R script available for visualising CNVs by chromosome (iii) Input format: BAM

Atlas2	[85]	https://www.hgsc.bcm.edu/software/atlas-2	(i) Calls SNPs and indels for WES data (ii) Requires BAM file as input (iii) Output: VCF format

GATK, SOAPsnp, and SAMTools have constantly been cited in large genetic association projects indicating their ease of use, reliability, and functionality. However, this is also helped by the fact that they have additional features. There are other tools such as Beagle [68], IMPUTE2 [86], and MaCH [87] which have modules for SNP and genotype calling but are mostly used for their main purpose such as imputation and haplotype phasing.

**Table 3:** Tools for predicting variant effects, identifying neutral and pathogenic mutations.

Name	Reference	MCC	Comments
^*^SIFT	[88, 89]	0.30 (unweighted)	It is a highly cited with many projects using and citing it since 2001, uses available evolutionary information and is continually updated, is easy to use through VEP, and provides two classifications: “deleterious” and “tolerated.”

^*^PolyPhen-2	[42]	0.43	It provides a high quality multiple sequence alignment pipeline and is optimized for high-throughput analysis of NGS data, is cited and used by many projects of different types, is easy to use through VEP, and provides three classifications: “probably damaging,” “possibly damaging,” and “benign.”

^*^FATHMM	[43]	0.72	It is a high performing prediction tool. Clear examples are available on the website. It offers flexibility to the user for weighted (trained using inherited disease causing mutations) and unweighted (conservation-based approach) predictions and also offers protein domain-phenotype association information, and has options for cancer-specific predictions (FATHMM-Cancer) and predictions for noncoding variants (FATHMM-MKL).

GERP++ (and GERP)	[90–92]	N/A	It determines constrained elements within the human genome; therefore variants in them are likely to induce functional changes. Can provide unique details about the candidate variant(s).

PhyloP	[93]	N/A	It helps detect nonneutral substitutions, similar aim with GERP.

CADD	[11]	—	It provides annotation and scores for all variants in the genome considering a wide range of biological features.

GWAVA	[13]	—	It provides predictions for variants in the noncoding part of the genome.

^*^SNAP	[94]	0.47	It predicts the effects of nonsynonymous polymorphisms and is cited and used many times and should be used to check whether the predicted effect is matched by the putative causal variant. However it was labelled “too slow” for high throughput analyses by [46].

PupaSuite	[95]	—	It identifies functional SNPs using the SNPeffect [96] database and evolutionary information.

Mutation Assessor-2	[97]	—	It predicts the impact of protein mutations and is user friendly website and accepts many formats.

^*^PANTHER	[98, 99]	0.53 (unweighted)	It predicts the effect of amino acid change based on protein evolutionary relationships. It provides a number ranging from 0 (neutral) to −10 (most likely deleterious) and allows the user to decide on the “deleteriousness” threshold. It is constantly updated making it a very reliable tool.

CONDEL-2	[45]	—	It combines FATHMM and Mutation Assessor (as of version 2) in order to improve prediction. It theoretically outperforms the tools it is using in comparison to when the tools are used individually.

^*^MutPred	[44]	0.63	It predicts whether a missense mutation is going to be harmful or not based on a variety of features such as sequence conservation, protein structure, and functional annotations and is praised in recent comparative study by [46].

^*^SNPs&GO	[100]	0.65	It is reported to have performed best amongst many prediction tools in [46] and provides two classifications: “disease related” and “neutral.”

Human Splicing Finder	[47]	N/A	It predicts the effect of noncoding variants in terms of alteration of splicing. Useful for compound heterozygotes if one allele is intronic.

Others	[101–104]	0.19 0.43 0.40 —	^*^nsSNPAnalyzer (requires 3D structure coordinates), ^*^PhD SNP, ^*^Polyphen (not supported any more), and PMUT

Many methods have been developed to predict the functional effect of variants in the genome. Many of the tools listed above use different features and datasets to predict these effects. This is not an exhaustive list of all prediction tools but a collection of the most used/cited ones.

^*^Comprehensive information about the prediction tool including accuracy, specificity, and sensitivity available in [43, 46]. N/A: not applicable. MCC: Matthew's correlation coefficient. MCCs are obtained from [43].

**Table 4:** What is needed for a genetic study?

Material	Notes
“Sufficient” number of high-quality sequencing/genotype data	Amount needed can vary from one proband and a few family members (for very rare Mendelian disorders) to thousands of cases and controls (for certain common complex disorder/traits)

List of candidate genes	Websites such as http://omim.org/ and http://ghr.nlm.nih.gov/; and software such as SNPs3D can be helpful

Identification of variant calling tool	Such as in Table 2

Identification of variant effect predictor tool	Such as in Table 3; tools usually require conversion of VCF to VEP format (Ensembl website)

Knowledge of human population variation databases	That is, HapMap, 1000 Genomes Project, EVS, dbSNP, and internal databases

Knowledge of databases storing information about genes and their products	That is, OMIM, Gene (NCBI), GeneCards, Unigene (NCBI), GEO Profiles (NCBI), HomoloGene (NCBI), and Mouse knockout databases (such as http://www.informatics.jax.org/, http://www.tigm.org/database/ and http://www.nc3rs.org.uk/category.asp?catID=8). Search the literature using PubMed and/or Web of Science.

The most important factors when carrying out a genetic association study are (i) the availability of reliable data (ii) bioinformatics and biological expertise, and (iii) careful planning.

## References

[1] Danecek P., Auton A., Abecasis G. (2011). The variant call format and VCFtools. *Bioinformatics*.

[2] McKenna A., Hanna M., Banks E. (2010). The genome analysis toolkit: a MapReduce framework for analyzing next-generation DNA sequencing data. *Genome Research*.

[3] Metzker M. L. (2010). Sequencing technologies—the next generation. *Nature Reviews Genetics*.

[4] Bonetta L. (2010). Whole-genome sequencing breaks the cost barrier. *Cell*.

[5] Pettersson E., Lundeberg J., Ahmadian A. (2009). Generations of sequencing technologies. *Genomics*.

[6] Hedges D. J. (2011). Comparison of three targeted enrichment strategies on the SOLiD sequencing platform. *PLoS ONE*.

[7] Teer J. K., Mullikin J. C. (2010). Exome sequencing: the sweet spot before whole genomes. *Human Molecular Genetics*.

[8] Bick D., Dimmock D. (2011). Whole exome and whole genome sequencing. *Current Opinion in Pediatrics*.

[9] Choi M., Scholl U. I., Ji W. (2009). Genetic diagnosis by whole exome capture and massively parallel DNA sequencing. *Proceedings of the National Academy of Sciences of the United States of America*.

[10] The ENCODE Project Consortium (2004). The ENCODE (ENCyclopedia Of DNA Elements) project. *Science*.

[11] Kircher M., Witten D. M., Jain P., O'Roak B. J., Cooper G. M., Shendure J. (2014). A general framework for estimating the relative pathogenicity of human genetic variants. *Nature Genetics*.

[12] Shihab H. A., Rogers M. F., Gough J. (2015). An integrative approach to predicting the functional effects of non-coding and coding sequence variation. *Bioinformatics*.

[13] Ritchie G. R., Dunham I., Zeggini E., Flicek P. (2014). Functional annotation of noncoding sequence variants. *Nature Methods*.

[14] Kiezun A., Garimella K., Do R. (2012). Exome sequencing and the genetic basis of complex traits. *Nature Genetics*.

[15] Ku C.-S., Naidoo N., Pawitan Y. (2011). Revisiting Mendelian disorders through exome sequencing. *Human Genetics*.

[16] Gilissen C., Hoischen A., Brunner H. G., Veltman J. A. (2012). Disease gene identification strategies for exome sequencing. *European Journal of Human Genetics*.

[17] The 1000 Genomes Project Consortium (2010). A map of human genome variation from population-scale sequencing. *Nature*.

[18] Brandstätter A., Sänger T., Lutz-Bonengel S. (2005). Phantom mutation hotspots in human mitochondrial DNA. *Electrophoresis*.

[19] Alsaadi M. M., Gaunt T. R., Boustred C. R. (2012). From a single whole exome read to notions of clinical screening: primary ciliary dyskinesia and RSPH9 p.Lys268del in the Arabian Peninsula. *Annals of Human Genetics*.

[20] Castleman V. H., Romio L., Chodhari R. (2008). Mutations in radial spoke head protein genes *RSPH9* and *RSPH4A* cause primary ciliary dyskinesia with central-microtubular-pair abnormalities. *American Journal of Human Genetics*.

[21] Gnirke A., Melnikov A., Maguire J. (2009). Solution hybrid selection with ultra-long oligonucleotides for massively parallel targeted sequencing. *Nature Biotechnology*.

[22] Bainbridge M. N., Wang M., Burgess D. L. (2010). Whole exome capture in solution with 3 Gbp of data. *Genome Biology*.

[23] Sulonen A.-M., Ellonen P., Almusa H. (2011). Comparison of solution-based exome capture methods for next generation sequencing. *Genome biology*.

[24] Chan E. Y., Komar A. A. (2009). Next-generation sequencing methods: impact of sequencing accuracy on SNP discovery. *Single Nucleotide Polymorphisms*.

[25] Ye K., Schulz M. H., Long Q., Apweiler R., Ning Z. (2009). Pindel: a pattern growth approach to detect break points of large deletions and medium sized insertions from paired-end short reads. *Bioinformatics*.

[26] Thorvaldsdóttir H., Robinson J. T., Mesirov J. P. (2013). Integrative genomics viewer (IGV): high-performance genomics data visualization and exploration. *Briefings in Bioinformatics*.

[27] Li H., Handsaker B., Wysoker A. (2009). The Sequence Alignment/Map format and SAMtools. *Bioinformatics*.

[28] Liu X., Han S., Wang Z., Gelernter J., Yang B.-Z. (2013). Variant callers for next-generation sequencing data: a comparison study. *PLoS ONE*.

[29] Purcell S., Neale B., Todd-Brown K. (2007). PLINK: a tool set for whole-genome association and population-based linkage analyses. *The American Journal of Human Genetics*.

[30] Woods C. G., Valente E. M., Bond J., Roberts E. (2004). A new method for autozygosity mapping using single nucleotide polymorphisms (SNPs) and EXCLUDEAR. *Journal of Medical Genetics*.

[31] Carr I. M., Bhaskar S., O' Sullivan J. (2013). Autozygosity Mapping with Exome Sequence Data. *Human Mutation*.

[32] Carr I. M., Flintoff K. J., Taylor G. R., Markham A. F., Bonthron D. T. (2006). Interactive visual analysis of SNP data for rapid autozygosity mapping in consanguineous families. *Human Mutation*.

[33] Stenson P. D., Ball E. V., Mort M., Phillips A. D., Shaw K., Cooper D. N. (2012). UNIT 1.13 The Human Gene Mutation Database (HGMD) and its exploitation in the fields of personalized genomics and molecular evolution. *Current Protocols in Bioinformatics*.

[34] Landrum M. J., Lee J. M., Riley G. R. (2014). ClinVar: public archive of relationships among sequence variation and human phenotype. *Nucleic Acids Research*.

[35] Fokkema I. F. A. C., Taschner P. E. M., Schaafsma G. C. P., Celli J., Laros J. F. J., den Dunnen J. T. (2011). LOVD v.2.0: the next generation in gene variant databases. *Human Mutation*.

[36] http://omim.org/.

[37] McCarthy D. J., Humburg P., Kanapin A. (2014). Choice of transcripts and software has a large effect on variant annotation. *Genome Medicine*.

[38] Ng S. B., Turner E. H., Robertson P. D. (2009). Targeted capture and massively parallel sequencing of 12 human exomes. *Nature*.

[39] Alsaadi M. M., Erzurumluoglu A. M., Rodriguez S. (2014). Nonsense mutation in coiled-coil domain containing 151 gene (CCDC151) causes primary ciliary dyskinesia. *Human Mutation*.

[40] Ng P. C., Henikoff S. (2006). Predicting the effects of amino acid substitutions on protein function. *Annual Review of Genomics and Human Genetics*.

[41] Ng P. C., Henikoff S. (2003). SIFT: predicting amino acid changes that affect protein function. *Nucleic Acids Research*.

[42] Adzhubei I. A., Schmidt S., Peshkin L. (2010). A method and server for predicting damaging missense mutations. *Nature Methods*.

[43] Shihab H. A., Gough J., Cooper D. N. (2013). Predicting the functional, molecular, and phenotypic consequences of amino acid substitutions using hidden Markov models. *Human Mutation*.

[44] Li B., Krishnan V. G., Mort M. E. (2009). Automated inference of molecular mechanisms of disease from amino acid substitutions. *Bioinformatics*.

[45] González-Pérez A., López-Bigas N. (2011). Improving the assessment of the outcome of nonsynonymous SNVs with a consensus deleteriousness score, Condel. *The American Journal of Human Genetics*.

[46] Thusberg J., Olatubosun A., Vihinen M. (2011). Performance of mutation pathogenicity prediction methods on missense variants. *Human Mutation*.

[47] Desmet F.-O., Hamroun D., Lalande M., Collod-Bëroud G., Claustres M., Béroud C. (2009). Human Splicing Finder: an online bioinformatics tool to predict splicing signals. *Nucleic Acids Research*.

[48] McLaren W., Pritchard B., Rios D., Chen Y., Flicek P., Cunningham F. (2010). Deriving the consequences of genomic variants with the ensembl API and SNP effect predictor. *Bioinformatics*.

[49] Shihab H. A., Gough J., Cooper D. N., Day I. N. M., Gaunt T. R. (2013). Predicting the functional consequences of cancer-associated amino acid substitutions. *Bioinformatics*.

[50] Capriotti E., Altman R. B. (2011). A new disease-specific machine learning approach for the prediction of cancer-causing missense variants. *Genomics*.

[51] Sauna Z. E., Kimchi-Sarfaty C. (2011). Understanding the contribution of synonymous mutations to human disease. *Nature Reviews Genetics*.

[52] Buske O. J., Manickaraj A., Mital S., Ray P. N., Brudno M. (2013). Identification of deleterious synonymous variants in human genomes. *Bioinformatics*.

[53] Day I. N. M. (2010). dbSNP in the detail and copy number complexities. *Human Mutation*.

[54] Musumeci L., Arthur J. W., Cheung F. S. G., Hoque A., Lippman S., Reichardt J. K. V. (2010). Single Nucleotide Differences (SNDs) in the dbSNP database may lead to errors in genotyping and haplotyping studies. *Human Mutation*.

[55] Inglis P. N., Boroevich K. A., Leroux M. R. (2006). Piecing together a ciliome. *Trends in Genetics*.

[56] Franceschini A., Szklarczyk D., Frankild S. (2013). STRING v9.1: protein-protein interaction networks, with increased coverage and integration. *Nucleic Acids Research*.

[57] Kanehisa M., Goto S. (2000). KEGG: kyoto encyclopedia of genes and genomes. *Nucleic Acids Research*.

[58] Yue P., Melamud E., Moult J. (2006). SNPs3D: candidate gene and SNP selection for association studies. *BMC Bioinformatics*.

[59] Marques-Pinheiro A., Marduel M., Rabès J.-P. (2010). A fourth locus for autosomal dominant hypercholesterolemia maps at 16q22.1. *European Journal of Human Genetics*.

[60] Audrézet M.-P., Chen J.-M., Raguénès O. (2004). Genomic rearrangements in the CFTR gene: extensive allelic heterogeneity and diverse mutational mechanisms. *Human Mutation*.

[61] Zheng X. L., Sadler J. E. (2008). Pathogenesis of thrombotic microangiopathies. *Annual Review of Pathology: Mechanisms of Disease*.

[62] Sobreira N. L. M., Cirulli E. T., Avramopoulos D. (2010). Whole-genome sequencing of a single proband together with linkage analysis identifies a Mendelian disease gene. *PLoS Genetics*.

[63] Norio R. (2003). Finnish disease heritage I: characteristics, causes, background. *Human Genetics*.

[64] Norio R. (2003). Finnish Disease Heritage II: population prehistory and genetic roots of Finns. *Human Genetics*.

[65] Norio R. (2003). The finnish disease heritage III: the individual diseases. *Human Genetics*.

[66] Woods C. G., Cox J., Springell K. (2006). Quantification of homozygosity in consanguineous individuals with autosomal recessive disease. *American Journal of Human Genetics*.

[67] Williams A. L., Patterson N., Glessner J., Hakonarson H., Reich D. (2012). Phasing of many thousands of genotyped samples. *The American Journal of Human Genetics*.

[68] Browning S. R., Browning B. L. (2007). Rapid and accurate haplotype phasing and missing-data inference for whole-genome association studies by use of localized haplotype clustering. *The American Journal of Human Genetics*.

[69] Blumenthal M. N. (2012). Genetic, epigenetic, and environmental factors in asthma and allergy. *Annals of Allergy, Asthma & Immunology*.

[70] Kettunen J., Tukiainen T., Sarin A.-P. (2012). Genome-wide association study identifies multiple loci influencing human serum metabolite levels. *Nature Genetics*.

[71] Combarros O., Cortina-Borja M., Smith A. D., Lehmann D. J. (2009). Epistasis in sporadic Alzheimer's disease. *Neurobiology of Aging*.

[72] Farooqi S., Rau H., Whitehead J., O'Rahilly S. (1998). *ob* gene mutations and human obesity. *Proceedings of the Nutrition Society*.

[73] Homer N., Merriman B., Nelson S. F. (2009). Local alignment of two-base encoded DNA sequence. *BMC Bioinformatics*.

[74] Langmead B., Salzberg S. L. (2012). Fast gapped-read alignment with Bowtie 2. *Nature Methods*.

[75] Stitziel N. O., Kiezun A., Sunyaev S. R. (2011). Computational and statistical approaches to analyzing variants identified by exome sequencing. *Genome Biology*.

[76] Li H., Durbin R. (2009). Fast and accurate short read alignment with Burrows-Wheeler transform. *Bioinformatics*.

[77] Li H., Ruan J., Durbin R. (2008). Mapping short DNA sequencing reads and calling variants using mapping quality scores. *Genome Research*.

[78] Li R., Yu C., Li Y. (2009). SOAP2: an improved ultrafast tool for short read alignment. *Bioinformatics*.

[79] Le S. Q., Durbin R. (2011). SNP detection and genotyping from low-coverage sequencing data on multiple diploid samples. *Genome Research*.

[80] Quinlan A. R., Stewart D. A., Strömberg M. P., Marth G. T. (2008). Pyrobayes: an improved base caller for SNP discovery in pyrosequences. *Nature Methods*.

[81] Li R., Li Y., Fang X. (2009). SNP detection for massively parallel whole-genome resequencing. *Genome Research*.

[82] Li D., Guo Y., Shao H. (2010). Genetic diversity, molecular phylogeny and selection evidence of the silkworm mitochondria implicated by complete resequencing of 41 genomes. *BMC Evolutionary Biology*.

[83] Li S., Wang S., Deng Q. (2012). Identification of genome-wide variations among three elite restorer lines for hybrid-rice. *PLoS ONE*.

[84] Boeva V., Popova T., Bleakley K. (2012). Control-FREEC: a tool for assessing copy number and allelic content using next-generation sequencing data. *Bioinformatics*.

[85] Challis D., Yu J., Evani U. S. (2012). An integrative variant analysis suite for whole exome next-generation sequencing data. *BMC Bioinformatics*.

[86] Howie B., Marchini J., Stephens M. (2011). Genotype imputation with thousands of genomes. *G3: Genes, Genomes, Genetics*.

[87] Li Y., Willer C. J., Ding J., Scheet P., Abecasis G. R. (2010). MaCH: using sequence and genotype data to estimate haplotypes and unobserved genotypes. *Genetic Epidemiology*.

[88] Sim N.-L., Kumar P., Hu J., Henikoff S., Schneider G., Ng P. C. (2012). SIFT web server: predicting effects of amino acid substitutions on proteins. *Nucleic Acids Research*.

[89] Kumar P., Henikoff S., Ng P. C. (2009). Predicting the effects of coding non-synonymous variants on protein function using the SIFT algorithm. *Nature Protocols*.

[90] Davydov E. V., Goode D. L., Sirota M., Cooper G. M., Sidow A., Batzoglou S. (2010). Identifying a high fraction of the human genome to be under selective constraint using GERP++. *PLoS Computational Biology*.

[91] Cooper G. M., Goode D. L., Ng S. B. (2010). Single-nucleotide evolutionary constraint scores highlight disease-causing mutations. *Nature Methods*.

[92] Cooper G. M., Stone E. A., Asimenos G., Green E. D., Batzoglou S., Sidow A. (2005). Distribution and intensity of constraint in mammalian genomic sequence. *Genome Research*.

[93] Pollard K. S., Hubisz M. J., Rosenbloom K. R., Siepel A. (2010). Detection of nonneutral substitution rates on mammalian phylogenies. *Genome Research*.

[94] Bromberg Y., Rost B. (2007). SNAP: predict effect of non-synonymous polymorphisms on function. *Nucleic Acids Research*.

[95] Conde L., Vaquerizas J. M., Dopazo H. (2006). PupaSuite: finding functional single nucleotide polymorphisms for large-scale genotyping purposes. *Nucleic Acids Research*.

[96] Reumers J., Schymkowitz J., Ferkinghoff-Borg J., Stricher F., Serrano L., Rousseau F. (2005). SNPeffect: a database mapping molecular phenotypic effects of human non-synonymous coding SNPs. *Nucleic Acids Research*.

[97] Reva B., Antipin Y., Sander C. (2011). Predicting the functional impact of protein mutations: application to cancer genomics. *Nucleic Acids Research*.

[98] Thomas P. D., Kejariwal A., Campbell M. J. (2003). PANTHER: a browsable database of gene products organized by biological function, using curated protein family and subfamily classification. *Nucleic Acids Research*.

[99] Mi H., Dong Q., Muruganujan A., Gaudet P., Lewis S., Thomas P. D. (2009). PANTHER version 7: improved phylogenetic trees, orthologs and collaboration with the Gene Ontology Consortium. *Nucleic Acids Research*.

[100] Calabrese R., Capriotti E., Fariselli P., Martelli P. L., Casadio R. (2009). Functional annotations improve the predictive score of human disease-related mutations in proteins. *Human Mutation*.

[101] Bao L., Zhou M., Cui Y. (2005). nsSNPAnalyzer: identifying disease-associated nonsynonymous single nucleotide polymorphisms. *Nucleic Acids Research*.

[102] Capriotti E., Calabrese R., Casadio R. (2006). Predicting the insurgence of human genetic diseases associated to single point protein mutations with support vector machines and evolutionary information. *Bioinformatics*.

[103] Ramensky V., Bork P., Sunyaev S. (2002). Human non-synonymous SNPs: server and survey. *Nucleic Acids Research*.

[104] Ferrer-Costa C., Gelpí J. L., Zamakola L., Parraga I., de la Cruz X., Orozco M. (2005). PMUT: a web-based tool for the annotation of pathological mutations on proteins. *Bioinformatics*.

