# Supplementary material for: Identifying Highly Penetrant Disease Causal Mutations Using Next Generation Sequencing: Guide to Whole Process
Source: Biomed Res Int. 2015 Apr 6;2015:923491. doi: 10.1155/2015/923491 (PMC4461748; doi:10.1155/2015/923491)
Supplement: Supplementary file 1 — S1: List of useful bioinformatics command, parameter and files for variant calling, annotation and analysis. Figure S1: Pedigree trees demonstrating examples of relevant consanguineous unions. Figure S2: Example of the routes taken when analysing complex disorders. [file 923491.f1.zip › 923491.f1/923491.f1 (1).docx]

###### **Supp. Material and Methods**

These commands are here to guide the user. However where complications arise, other options may have to be included thus requires reading documentation provided by the bioinformatics tools.

For users who are not familiar with UNIX commands and programming languages, the Galaxy server (https://usegalaxy.org/) provides a user-friendly interface by providing 'push-button' features for use for the NGS read QC, NGS read alignment, variant calling and variant annotation stages. Many other related UNIX-based features (e.g. Text manipulation, Filter and Sort) have also been adapted for use with the click of a button.

For more advanced pipelines and documentation, Biostars (https://www.biostars.org/) provides a very useful medium where questions and answers are exchanged amongst bioinformaticians.

**Parameters used in BWA for read alignments:** bwa aln -o 1 -e 50 -m 10000 -t 4 -i 15 -q 10 -I

*-I at the end is for Illumina NGS platforms*

**Parameters used in GATK (for SNPs):** java -jar GenomeAnalysisTK.jar -T UnifiedGenotyper -stand_call_conf 50 -stand_emit_conf 10.0 -A DepthOfCoverage -A RMSMappingQuality -baq CALCULATE_AS_NECESSARY

**Parameters used in GATK (for InDels):** java -jar GenomeAnalysisTK.jar -T UnifiedGenotyper -stand_call_conf 50 -stand_emit_conf 10.0 -A DepthOfCoverage -A RMSMappingQuality -baq CALCULATE_AS_NECESSARY -glm INDEL

**Obtaining Ensembl VEP annotations for VCFs (including SIFT, Polyphen and Condel predictions):**

1. Download latest package (and *plugins) from Ensembl website: ([www.ensembl.org/info/docs/variation/vep/index.html](http://www.ensembl.org/info/docs/variation/vep/index.html))
2. Tar xvf downloaded file(s)
3. perl INSTALL.pl – and download *Homo sapiens* cache(s)
4. perl variant_effect_predictor.pl -i **file.vcf** -o **file.vep** --protein --cache --regulatory --gmaf --force_overwrite --sift b --polyphen b --plugin Condel,/data/home/~/ensembl-tools-release-75/scripts/variant_effect_predictor/ensembl-variation-VEP_plugins-e6cec6a/config/Condel/config,b --fork 8 --canonical --individual all --pubmed --maf_esp --symbol

*to use Condel plugin:

1- Download latest Ensembl plugins from: <https://github.com/ensembl-variation/VEP_plugins>

2- tar -xvf downloaded file

2- mv Condel.pm ~/.vep/Plugins (create Plugins folder if not there; also .vep is a hidden folder)

3- edit the condel_SP.conf file (in config/Condel/config/) and set the 'condel.dir' parameter to /data/home/~/variant_effect_predictor/ensembl-variation-VEP_plugins-e6cec6a/config/Condel

**Example of commands used to filter variants in VEP file:** To grab list of all rare/unique and homozygous mutations in candidate genes: grep -f **Candidate_genes.txt** **file.vep** | grep -f **PHI_SO_terms.txt** | grep CANONICAL | grep HOM | grep _[A-Z]/ > **file_candidate_mutations.txt**

or use grep GMAF=[A-Z]:0.00 instead of grep _[A-Z]/ for variants which are present in the 1000GP but rare

**Files used:**

**Candidate_genes.txt:** a text file containing Ensembl IDs of your candidate genes – one per row

**PHI_SO_terms.txt:** a text file containing VEP SO terms which would be classified as a Φ mutation (available as Supp. File)

**Command for Autozygosity plotting in AutoZplotter:** python autozplotter.py

**Parameters used for Autozygosity mapping in Plink:** plink --file <ped/map> --homozyg --noweb --homozyg-window-kb 1000 --homozyg-window-het 1 --homozyg-group --out <output>

**PHI_SO_terms.txt**

#Ensembl VEP sequence ontology (SO) terms of predicted high impact (PHI) mutations (see Alsaadi and Erzurumluoglu *et al*, 2014 for example) - SO terms correct as of March 2015

#Can be used when 'greping' out PHI mutations from Ensembl VEP annotated VCF files (command: grep -f PHI_SO_terms.txt file_name.vep)

splice_acceptor_variant

splice_donor_variant

stop_lost

frameshift_variant

transcript_ablation

stop_gained

missense_variant

initiator_codon_variant

inframe_insertion

inframe_deletion

Union of **double (first) cousins**, where both parents share the same four grandparents

*F* = 0.125

Union between **first cousins**, a very common form of consanguineous unions

*F* = 0.0625

**Supp. Figure S1** Consanguineous Unions where F ≥ 0.0625

**Uncle-niece unions** are common in Southern India, an uncommon type anywhere else (Aunt-nephew unions is also consanguineous, and their offspring would be expected to have similar *F* value)

*F* = 0.125

**Supp. Figure S2: Finding ‘the lot’ in Complex disorders**

**All (QC checked) variants from sample/exomes**

Is the disorder/trait dichotomous or quantitative?

**Quantitative**

**Dichotomous**

**Compare case and controls and carry out linear regression**

- Protective or risk effect?

**Compare case and controls and carry out logistic regression**

- Protective or risk allele?

Reach exome-wide significance level?

**No**

**Yes**

Consider the clinical relevance of variant

**Replicate**

Most ideally in an independent dataset

**(i) Use collapsing methods**

Combining multiple rare variants to give a single gene-based variable

Replicated?

***and/or***

***and/or***

**(ii) Carry out meta-analysis**

**No**

**Yes**

**Publish**

*Return to Figure 2 and re-check assumptions*

**(iii) Sequence more exomes**

**Supp. Figure S2 Finding ‘the lot’ in Complex disorders: Searching for causal variants (using a WES example)**

The standard procedure is to compare cases with controls and detect whether there are any significant differences in the allele frequencies of each variant. The statistical power of this approach is going to predominantly depend on sample size and penetrance of the causal variant. Covariates should be identified and population stratification should be controlled for in the regression models. The clinical significance of the variant must also be taken into account especially when searching for variants with very low effect sizes. One must consider whether it is worth sequencing more exomes in order to reach exome wide (or genome-wide) significance for the identification of a variant which does not have any considerable effect on patients’ health. This figure is here to serve as an example and by no means reflects an exhaustive model – there are many alternative routes researchers can take to identify common-complex disease causal variants.
